# Pro: Are we ready to translate Alzheimer's disease modifying therapies to people with Down syndrome?

**DOI:** 10.1186/s13195-014-0060-7

**Published:** 2014-09-12

**Authors:** Michael S Rafii

**Affiliations:** 1 Department of Neurosciences, University of California, San Diego, 9500 Gilman Drive, MC 0949, La Jolla 92093, CA, USA

## Abstract

**Background:**

Down Syndrome (DS) is caused by trisomy of chromosome 21, which includes the gene for the amyloid precursor protein (APP) and leads to overproduction of beta-amyloid. Clinical-pathological studies indicate that individuals with DS begin demonstrating Alzheimer’s disease (AD) pathology during adolescence and that 100% exhibit such changes by age 40. Individuals with DS therefore represent a highly enriched population for AD. Additionally, owing to their baseline intellectual disability, people with DS represent a more vulnerable group of individuals as compared with other populations. Given the recent developments in AD biomarkers, combined with the prospect of achieving greater efficacy with earlier therapeutic intervention, it is logical to include adults with DS in prevention trials for AD.

**Discussion:**

The US Food and Drug Administration has released draft guidance on drug development for early-stage AD, based on the understanding that AD is a progressive disease with symptoms developing decades after the disease process has begun. New biomarkers now permit detection of AD pathology in asymptomatic individuals such that there now exists an opportunity to conduct clinical trials of potentially disease-modifying drugs in the earliest stages of the disease and perhaps have the greatest chance of demonstrating efficacy. As such, clinical trials are being actively planned or conducted in individuals with causative mutations in the APP, presenilin-1 (*PSEN1*), and presenilin-2 (*PSEN2*) genes.

**Summary:**

Individuals with DS comprise perhaps the largest group of people with genetically determined AD, with a worldwide population of about 6 million people. Only by inclusion can we provide access to rational therapies that offer the greatest chance of benefiting this highly at-risk population.

## Introduction

### Preclinical Alzheimer’s disease

Converging evidence from longitudinal studies of clinically normal older and familial Alzheimer’s disease (AD) cohorts strongly suggests that the AD pathophysiological process begins decades before the manifestation of clinical dementia. Recent advances in biomarkers, including amyloid positron emission tomography (PET), Tau PET, and volumetric magnetic resonance imaging (MRI), and cerebrospinal fluid studies permit presymptomatic detection of AD pathology. Hypothetical models have been proposed in which these biomarkers become increasingly abnormal in an ordered manner as the disease progresses and symptoms emerge [1]. Longitudinal studies of familial AD have provided substantial support for and refinement of such models [2],[3].

Mutations of all genes known to be involved in familial AD - presenilin-1 (*PSEN1*), *PSEN2*, and amyloid precursor protein (*APP*) - contribute to increased absolute or relative production of the 42-amino-acid-length cleavage product of APP, beta-amyloid (Aβ), which is the main constituent of the amyloid plaques that characterize AD [4]. This tight link between genetic determinants of AD and the overproduction of Aβ is compelling support for the amyloid hypothesis and has been the focal point in the development of disease-modifying drugs for AD.

Presumably, disease-modifying treatments should begin prior to the onset of cognitive symptoms and before extensive neurodegeneration has occurred. A decade of negative anti-amyloid trials in mild to moderate AD supports the notion that disease-modifying treatment in the dementia stage may simply be too late. The field of AD drug development has therefore steadily marched earlier and earlier in the disease continuum, evaluating anti-amyloid treatments in prodromal AD and, most recently, in preclinical AD. Although autosomal dominant mutation carriers represent less than 2% of all AD cases, such cohorts are now being intensely studied in secondary prevention trials of AD using anti-amyloid drugs, given the opportunity they afford for presymptomatic intervention.

## Discussion

### Down syndrome is preclinical Alzheimer’s disease

Down syndrome (DS), or trisomy 21, affects 400,000 people in the US and has an incidence of 1 out of 691 live births [5]. It is caused by meiotic non-disjunction, leading to an extra copy of chromosome 21, on which the APP gene resides. Consequently, there is increased APP mRNA and protein expression as well as higher levels of Aβ [6]. Because of this overproduction of Aβ, by the age of 40, virtually all people with DS show the same neuropathological changes as is seen in AD [7]. Moreover, the cholinergic losses seen in the brains of individuals with DS are identical to those observed in AD [8]. In contrast to sporadic AD, however, amyloid plaques and neurofibrillary tangles in DS start developing in people as early as 12 years of age [9].

Recent data also indicate that AD biomarker changes in DS are similar to those observed in familial and sporadic AD. There is a sixfold increase in plasma Aβ in individuals with DS as compared with age-matched non-DS individuals [10]. Results of amyloid PET imaging from individuals with DS are also consistent with those seen in non-DS individuals with AD [11]. And, as in familial and sporadic AD, the presence of the apolipoprotein E ɛ4 allele is associated with greater accumulation of Aβ protein in the brains of adults with DS [12] and greater risk of an earlier age of onset of dementia [13]. The link between triplication of the APP gene and subsequent overproduction of Aβ leading to AD dementia in DS is further supported by the case of a DS individual with partial trisomy of chromosome 21 who was disomic for the APP gene and who did not develop dementia or any AD pathology [14].

Postmortem studies also indicate that adults with DS have the same prominent pattern of cerebral atrophy involving the medial temporal lobe structures as has been reported in the early stages of familial and sporadic AD [15]. Volumetric MRI studies of age-related brain changes in DS demonstrate the same pattern of hippocampal-specific atrophy observed in AD. Furthermore, the hippocampal atrophy in DS correlates with changes in memory measures [16].

In DS, in which intellectual disability is lifelong, the question arises as to how changes in cognition and functioning can be reliably related to the emergence of AD symptomatology. It should be noted that baseline intellectual disability in DS is relatively static. Results from recent biomarker studies suggest that these techniques may be able to accurately discriminate progressive brain changes due to AD age-related changes in DS. Moreover, DS individuals who are over 40 years old show cognitive decline in somewhat discrete phases. Initially, there is an isolated, slowly progressive memory decline [17], analogous to the mild cognitive impairment stage of AD in the sporadic population, in which memory loss is the earliest neuropsychological deficit. This phase is followed by a decline in other cognitive functions and is coincident with functional decline and dementia onset [17]. Thus, older adults with DS represent a population in which the neuropsychological profile of cognitive change due to AD can be followed in conjunction with biomarker changes decades before dementia occurs.

### Imperative for Alzheimer’s disease prevention in Down syndrome

The number of older DS adults in the US is increasing. In fact, from 1979 through 2003, the prevalence of DS increased from 9.0 to 11.8 per 10,000 live births in 10 US regions [18]. This increase is due partially to the increase in the number of women who conceive after age 35 [19]. In addition, people with DS have experienced significant increases in life expectancy as a result of reduced institutionalization and improved access to medical care, such as surgical intervention for congenital heart defects [20]. With increased life expectancy, there has been an increase in the prevalence of older DS adults with dementia. It is currently estimated that there are over 200,000 people with DS over the age of 55 in the US [21]. Over 30% of DS adults more than 50 years old and over 50% of DS adults more than 60 years old have been diagnosed with AD dementia [22]. By the age of 70, approximately 75% of individuals with DS have dementia [23].

### Alzheimer’s disease prevention trials in Down syndrome

Given the high risk of people with DS developing AD, it is quite reasonable to consider prevention trials in this population. We have sufficient knowledge about AD to design and conduct secondary prevention studies in other genetically determined populations. Undeniably, there is more to learn about DS, but that does not preclude inclusion of this group in AD prevention trials in which the incidence and pathology of AD are well understood. One can envision a relatively small, early-stage study confirming feasibility and determining effect size in non-demented DS individuals to inform a larger late-stage trial. Variance in the rate of cognitive decline as well as signals on putative AD biomarkers in the initial phase of development would be used to calculate the sample size required, as well as the duration of treatment, for a definitive late-stage trial. Recent studies using amyloid PET imaging demonstrate that deposition of fibrillar amyloid is most dynamic between ages 35 and 55. This age range may be an appropriate window to assess anti-amyloid therapeutics.

Ultimately, however, endpoints in AD prevention trials in DS will be cognitive outcomes as these will have the greatest translatability to clinical meaningfulness. The US Food and Drug Administration recently provided guidance on drug development for the treatment of preclinical AD [24]. This guidance indicates that an effect on a valid and reliable cognitive assessment used as a single primary efficacy measure would be considered for approval in the context of a patient with positive biomarkers of AD [25]. Instruments will need to be sensitive for detecting change over time in relation to age, baseline performance, and other participant characteristics. Such valid cognitive assessment tools currently exist for DS [26]-[28].

## Summary

Multiple lines of evidence suggest that individuals with DS suffer exactly the same pathological process in later life as individuals with the other forms of AD. Indeed, there is little to distinguish the pathological changes in either condition. In DS, triplication of the APP gene leads to the overproduction of Aβ and drives amyloidogenic pathways leading to plaques, tangles, and neurodegeneration. Owing to the 100% prevalence of AD pathology in adults with DS, individuals with DS represent a well-defined subgroup of predetermined AD. With 6 million people worldwide, DS is the largest population of predictable AD cases. By including individuals with DS in prevention trials, there is an opportunity to provide access to potentially disease-altering therapies to this highly at-risk population.

## Abbreviations

Aβ: Beta-amyloid

AD: Alzheimer’s disease

APP: Amyloid precursor protein

DS: Down syndrome

MRI: Magnetic resonance imaging

PET: Positron emission tomography

## Competing interests

MSR has received research grants from Elan Corporation (Dublin, Ireland), Hoffmann-La Roche (Basel, Switzerland), Janssen Pharmaceuticals (Titusville, New Jersey), Genentech (South San Francisco, CA, USA), Eli Lilly and Company (Indianapolis, IN, USA), Accera (Broomfield, CO, USA), Merck (Whitehouse Station, NJ, USA), and Bristol-Myers Squibb (New York City, NY, USA). He has served as a consultant to Novartis (Basel, Switzerland).
